# Accreditation standards for medical imaging services

**DOI:** 10.4103/0971-3026.63042

**Published:** 2010-05

**Authors:** Zainab Zaidi

**Affiliations:** National Accreditation Board for Hospitals and Healthcare Providers (NABH), Quality Council of India, 2^nd^ floor, Bahadur Shah Zafar Marg, New Delhi-2, India

**Keywords:** Accreditation, medical imaging services, National Accreditation Board for Hospital and Healthcare Providers, Quality Council of India

## Abstract

The rapid changes in the healthcare system, with revolutionary advancements in imaging, along with the lack of any existing imaging standards in our country, have raised the need for an accreditation structure. The Quality Council of India (QCI) has therefore introduced standards for medical imaging services, focusing on the control of services, personnel, imaging processes and procedures, facility and environment, equipment, and documentation, as well as risk control and safety. This article deals briefly with the standards structured by the QCI for accreditation of imaging services.

## Introduction

Changing market forces, increasing health awareness, the advent of medical tourism and health insurance, and the entry of the corporate sector into health delivery have raised the demand for quality in healthcare services. Initial attempts were made by the Bureau of Indian Standards in the late 1980s, when the organization set down the standards for the voluntary accreditation of 30-, 100-, and 250-bedded hospitals. The National Institute of Health and Family Welfare specified rules for hospitals with more than 50 beds. Health is a state responsibility in India and therefore attempts have also been made in some states to frame standards for hospitals. However, such compartmentalized initiatives have led to further fragmentation of an already segmented sector. Realizing the need to establish a uniform accreditation structure across the country, which would suit Indian conditions and at the same time be credible in the eyes of international observers, an inter-ministerial taskforce was set up in 1991. As an outcome of its recommendations, the Quality Council of India (QCI) was established in 1997, as an autonomous body, with the mandate to establish and operate a national accreditation structure and to obtain international recognition for its accreditation schemes.

The National Accreditation Board for Hospitals and Healthcare Providers (NABH) was set up under the QCI to establish and operate accreditation programs for healthcare organizations. Diagnostic imaging forms an integral part of healthcare. The clinical advantages of these services are enormous and imaging affects critical decision-making at every stage of patient management. However, it could also represent an unnecessary cost to healthcare systems in the country, if the quality provided is less than optimal. The phenomenal growth in diagnostic imaging in the last two decades has further enhanced the importance of this field. NABH for healthcare takes into account some major aspects of imaging services in a hospital, although its role and scope are limited. Hence, to assess the quality and safety of imaging services and to have a method for the monitoring of quality standards, a basic accreditation program has been introduced in the country. These standards reflect the expectation of good services from the viewpoint of the service provider, the patient, and the referrer, as well as the safety regulatory bodies. These standards also take into consideration the requirements of safety and service standards of the Atomic Energy Regulatory Board (AERB) and the Pre-Natal Sex Determination (PNDT) regulations relevant to our country.

## Medical Imaging Services

The medical imaging services (MIS) cover investigations of patients that provide imaging information for the diagnosis, prevention, and treatment of disease or assessment of health. It includes conventional radiation-based diagnostic radiology as well as a wide variety of specialized techniques including USG, Doppler studies, bone densitometry, CT scan, MRI, *PET/CT*, *SPECT*, radionuclide imaging and therapy, and so on.

The MIS also include in its scope, the arrangements for requisition, patient identification, choice of appropriate (most informative and cost-effective) imaging techniques, patient information, patient consent, patient preparation, performance of imaging procedures, interpretation, reporting, and advice regarding the result, in addition to the consideration of safety and ethics in diagnostic imaging services.

## Scope of Services

Medical imaging standards are applicable to single and multiple service providers as well as hospital services. They include all modalities related to x-rays, fluoroscopy-based investigative and interventional procedures, mammography and related interventional procedures, bone mineral densitometry, USG, diagnostic as well as interventional, Doppler, MRI, CT scan, nuclear medicine, and procedures such as, radiofrequency ablation and laser / cryo / thermoablation.

## Brief About Standards

The standards have been grouped into six chapters dealing with all aspects of imaging services, from control of services, facilty and environment, imaging processes and procedures, equipment, documentation of records and control of risk and safety. Every chapter, after a brief description that explains its purpose, sets out a number of standards that need to be objectively assessed. The defined standards are patient-focused, cover the functions and systems of medical imaging as a whole, interventional as well as nuclear medicine service, and address the dimensions of quality, and support quality improvement. The standards take into account the need for all statutory compliance — AERB, Preconception and Prenatal Diagnostic Techniques (PC-PNDT), pollution control, and so on.

## Control of Services

The MIS standards emphasize the need to work collaboratively with clinical colleagues, to agree upon and select the appropriate imaging pathways to ensure diagnosis and / or treatment within a defined time frame, with minimal delays. The services have to be patient-focused and respectful to the patient and address their specific requirements. Proper attention has to be given to ensure the patients' privacy, dignity, and security. It is also essential to encourage the patients and their attendants to give feedback and to use this feedback for the betterment of the service delivery. The MIS, as per the guidelines, must provide a facility that is safe, clean, comfortable, and fit for the staff, patients, and others. The MIS has to ensure the provision of safe water, electricity, bio-waste and sewage disposal, and so on. The organization must have in place plans for the management of disasters and emergencies within the facility.

## Control of Imaging Processes and Procedures

The MIS have to ensure that all images are acquired and reported in accordance with the agreed protocols, by competent staff working within their defined scope of practice. Optimal diagnostic quality, with essential image characteristics, must be obtained in accordance with the best current practices. Interventional procedures must be conducted as per the defined protocols, by competent staff. It is also essential to ensure that drugs, isotopes, contrast media, and radiopharmaceuticals are stored, prescribed, prepared, and administered safely as per the statutory requirements. There have to be appropriate arrangements to deal with adverse reactions promptly, efficiently, and effectively.

## Control of Personnel

The management of staff has to be effective, fair, consistent, and supportive. The service contracts are to be managed effectively and efficiently to ensure high-quality care to patients. The organization should have a well-documented performance-appraisal system to be used as a tool for further development.

## Control of Equipment

The MIS has a duty to ensure appropriate procurement, installation, operation, maintenance, quality assurance, and replacement of all equipment, including ancillary equipment, such as, resuscitation equipment, protective clothing, and consumables. Records pertaining to the same should be maintained.

## Control of Documents and Records

The MIS have a duty to ensure that documents pertaining to policies, procedures, applicable laws and regulations, as well as guidelines for dealing with vulnerable patients (elderly, physically and / or mentally challenged, and children), obstetric patients, patients being administered sedation or anesthesia, and patients undergoing radiation, interventional or nuclear medicine services, are in place. It is the duty of the MIS to manage, store, and transfer all data in a secure manner, which must adhere to the statutory requirements (if any).

## Risk Control and Safety

The MIS have a duty to ensure that the organization has arrangements and general protection measures for staff, patients, and others, to restrict exposure to ionizing radiation and risks associated with the use of MRI, ablative and therapeutic devices, radionuclides, and so on. The organization has a duty to minimize and manage risk due to potentially violent and / or aggressive behavior in the facility. The management should promote a good and safe ambience, be prepared to manage adverse healthcare events, and minimize the risks associated with fire, electrocution, and other disasters.

## Summary

Accreditation of medical diagnostic services is the need of the hour. NABH accreditation for MIS is based on optimum standards and professional accountability, and encourages healthcare organizations to pursue excellence. The formulated standards have focused on statutory compliance, patient and staff safety, improved workflow, learning, self-development, improved performance, and reduction of risk. Its assessment relies on establishing management and technical competence in terms of accreditation standards, for delivering all the services within its scope. It goes beyond compliance and calls for the pursuit of excellence on a continuing basis.

